# Impact of protozoan cell death on parasite-host interactions and pathogenesis

**DOI:** 10.1186/1756-3305-3-116

**Published:** 2010-12-02

**Authors:** Carsten GK Lüder, Jenny Campos-Salinas, Elena Gonzalez-Rey, Ger van Zandbergen

**Affiliations:** 1 Institute for Medical Microbiology, Georg-August-University, Kreuzbergring 57, 37075 Göttingen, Germany; 2 Instituto de Parasitologia y Biomedicina "Lopez-Neyra", Parque Tecnologico Ciencias de la Salud, Avenida Conocimiento, s/n, 18100 Armilla (Granada), Spain; 3 Medical Microbiology and Hygiene, University of Ulm, Albert-Einstein-Allee 23, 89081 Ulm, Germany

## Abstract

PCD in protozoan parasites has emerged as a fascinating field of parasite biology. This not only relates to the underlying mechanisms and their evolutionary implications but also to the impact on the parasite-host interactions within mammalian hosts and arthropod vectors. During recent years, common functions of apoptosis and autophagy in protozoa and during parasitic infections have emerged. Here, we review how distinct cell death pathways in *Trypanosoma*, *Leishmania*, *Plasmodium *or *Toxoplasma *may contribute to regulation of parasite cell densities in vectors and mammalian hosts, to differentiation of parasites, to stress responses, and to modulation of the host immunity. The examples provided indicate crucial roles of PCD in parasite biology. The existence of PCD pathways in these organisms and the identification as being critical for parasite biology and parasite-host interactions could serve as a basis for developing new anti-parasitic drugs that take advantage of these pathways.

## Introduction

Surprisingly, the question of how protozoan parasites die was neglected for a long time. Death of unicellular organisms was generally assumed to occur in an uncoordinated manner, and the processes involved in life-or-death decisions after treatment of parasites with chemotherapeutic agents or after induction of anti-parasitic immunity were therefore largely ignored. This was mostly due to the assumption that genetically determined death pathways in single-celled organisms are not favourable during evolution. With the detection, however, of cell death markers characteristic for metazoan programmed cell death (PCD) in diverse free-living and parasitic protozoa their pathways to death became a topic of intense research.

Three main cell death pathways, i.e. apoptosis, autophagic cell death and necrosis are now being generally considered [1] and it has been recently proposed to adopt the criteria of this classification also for PCD in protozoa [2]. Signs of apoptosis have been recognized in divergent unicellular parasites including kinetoplastids, apicomplexans, *Trichomonas vaginalis*, *Giardia lamblia *and *Blastocystis hominis *[3]. Markers for apoptosis in protozoan parasites include cell shrinkage, chromatin condensation, DNA and nuclear fragmentation, loss of mitochondrial membrane potential (MMP) and translocation of phosphatidylserine (PS) from the inner to the outer leaflet of the plasma membrane, while the plasma membrane is not disrupted at least during early stages [4]. In contrast, necrosis typically includes cell and organelle swelling, loss of plasma membrane integrity and only moderate chromatin condensation. Death by necrosis has been described in trypanosomes [5,6]. It has also been suggested for blood stages of *Plasmodium *[7] although this is still a matter of debate [8,9]. It is important to note that necrosis can occur in a regulated and physiological manner [10,11] and that it is nowadays at least not generally considered an uncontrolled form of death. Autophagy is an evolutionary conserved process which is presumably present in all protozoan parasites [12]. It is thought to function primarily as a survival mechanism which is used to provide the cell with energy during stress conditions including starvation, for organelle turnover, or for remodelling a cell during differentiation. However, when adverse conditions take too long and exceed cellular capacity, they could promote autophagic cell death [13]. Autophagic cell death is thus defined as cell death that occurs in the context of autophagy [1] and has been described in several protozoan parasites [14-19]. The most important step in autophagy is the formation of a new membrane to engulf cellular material (cargo) to be digested; this membrane that eventually forms a double membrane-surrounded vesicle is called the autophagosome.

One of the main questions that emerge from the detection of PCD pathways in protozoan parasites is how we can exploit these processes to combat some of the most widespread and deadly infectious agents of humans and animals more efficiently. Surely, detailed knowledge of the death-inducing signals and environmental conditions, the underlying transduction pathways, and the death effectors of protozoan parasites are not only of major scientific interest but will open a treasure chest for the development of new anti-parasitic therapies. Another major prerequisite for exploiting protozoan PCD pathways is however a clear picture about the physiological implications of parasite PCD on the host-pathogen interaction and the course of disease. During recent years, several common themes emerged on the physiological functions of cell death pathways in protozoa. In the following, we discuss current knowledge on how parasite PCD might regulate parasite densities within the host, how it is involved in stress responses and differentiation of protozoan parasites, and how it modulates host immunity to infection. Where applicable, the molecular mechanisms which govern these processes are also reviewed.

### Regulation of parasite cell density by protozoan cell death

In order to establish sustained infections and transmission to new hosts, most parasites must avoid hyper-parasitism which would lead to the death of both the host and the parasite. Parasite numbers can be regulated by cell proliferation, cell cycle progression, or cell death. PCD in distinct protozoan parasites appears to determine cell densities at least under certain conditions (Table 1) and we hypothesize that it critically affects the parasite-host interaction by facilitating a sustained parasite-host equilibrium.

**Table 1:** Programmed cell death pathways and their possible roles in parasite biology and parasite-host interaction

Function	Parasite/Stage	Host	Form of death/Sign. pathway	Citations
**Density control**	*T. brucei*/short stumpy	mammal	apoptosis	[20,21]
	*T. brucei*/procyclic	tsetse fly	apoptosis	[22,23]
	*P. berghei*, *P. falciparum*/ookinetes	vector	apoptosis	[34,35]
	*T. cruzi*/epimastigotes	vector	apoptosis	[44,47]
	*T. cruzi*/amastigotes	mammal	apoptosis	[45,46]
**Immune silencing**	*L. major*/promastigotes	mammal	apoptosis	[60,61]
	*L. amazonensis*/amastigotes	mammal	apoptosis	[65]
	*T. cruzi*/trypomastigotes	vector, mammal	apoptosis	[66]
	*T. brucei*/short stumpy	mammal	apoptosis	[70]
	*Toxoplasma*/tachyzoites	mammal	apoptosis	[71]
**Differentiation**	*L. major*, *L. mexicana*	sand fly, mammal	autophagy	[75,76]
	*T. brucei*	mammal, sand fly	autophagy	[81]
	*T. cruzi*/epimastigotes	vector	autophagy	[84]
**Stress response**				
starvation	*T. brucei*	tsetse fly	autophagy	[87]
ROS, DHA, neuropeptides	*T. brucei*/blood stream form	mammal	autophagy/autophagic cell death	[15,16,87,90]
chemotherapeutic agents	*T. cruzi*/epimastigotes, trypomastigotes	vector, mammal	autophagy/cell death	[6,17,18,91-93]
starvation	*T. cruci*	vector	autophagy	[84]
heat shock	*L. infantum, L. donovani, L. amazonensis*	mammal	apoptosis	[97-101]

#### Apoptosis and cell density of African trypanosomes

*Trypanosoma brucei*, i.e. the causative agent of sleeping sickness in humans and of nagana in cattle can undergo apoptosis in the mammalian bloodstream form (BSF) [20,21] and the procyclic form within the midgut of the tsetse fly [22,23]. In the mammalian bloodstream, parasitemia of *T. brucei *increases and decreases periodically and this is partially due to effective antibody-mediated immune responses of the host and antigenic variation of the major surface glycoprotein of the parasite. However, the cell density of *T. brucei *is also regulated in axenic cultures in the absence of any host-derived immune effectors. After reaching a cell density-dependent threshold, further expansion of the cell population is restricted by differentiation from the replicating long slender (LS) form to the non-dividing short stumpy (SS) form [24]. Subsequently, the parasite density even decreases and this is accompanied by the occurrence of morphological and biochemical markers for apoptosis [20]. Likewise, cultivation of *T. brucei rhodesiense *under high-density conditions correlates with the appearance of parasites displaying an apoptotic phenotype [21]. Interestingly, Figarella and colleagues showed that cell death in BSF trypanosomes can also be induced *in vitro *by prostaglandin (PG) D_2 _and its metabolites of the J series in a dose-dependent manner, but not by PGE_2 _or PGF_2 _[20,25]. The IC_50 _of PGD_2_, i.e. the concentration necessary to inhibit cell growth of *T. brucei *BSF by 50% is 3.7 μM and this corresponds to the occurrence of 50% TUNEL-positive parasites after treatment with 5 μM PGD_2 _[20]. Trypanosomes produce PGs including PGD_2 _and secrete them into the environment [26]. In addition, levels of PGs increase in plasma and cerebrospinal fluid during human infections with *T. brucei *although it remains unknown whether these are mainly derived from the host or the parasite [27]. It is thus tempting to speculate that African trypanosomes sense the total level of both parasite-derived and host-derived PGD_2 _and its metabolites in their environment initiating a cell death program that subsequently leads to a reduction in parasite density. Such self-restriction would be probably particularly relevant during late-stage trypanosomiasis when parasites have invaded the central nervous system (CNS) and where PG levels are particularly elevated [27]. In addition, the immune defence against *T. brucei *is limited within the CNS and may therefore not suffice to restrict parasite numbers efficiently enough to favour a sustained infection. However, it might well be that PG-induced apoptosis also contributes to the restriction in parasite numbers in the peripheral blood.

After uptake of SS forms with the blood meal and differentiation to procyclic insect stages, the parasite load within the tsetse midgut remains remarkably constant despite their ability to divide [23]. This density regulation might be accomplished by apoptotic cell death as observed after treatment of procyclic *T. brucei rhodesiense in vitro *with the lectin concanavalin A [22,28]. Most trypanosomes of an infective blood meal indeed die within the midgut via a death process that display features of apoptosis and are consequently unable to establish within the midgut [29]. It was thus hypothesized that apoptotic cell death can regulate densities of procyclic trypanosomes in the tsetse midgut [23,30]. A correlation between parasite density and the level of apoptosis has however not been established yet. An important question also remains whether apoptosis in procyclic trypanosomes has evolved as a mechanism of parasite-initiated self-regulation or rather represents a pathway to death that is induced by immune factors of the insect vector. The latter view is supported by findings that parasite apoptosis appears to be regulated by lectins and possibly also antimicrobial peptides present in the midgut of tsetse flies [31,32]. Furthermore, antioxidants within the blood meal protect procyclic trypanosomes from undergoing apoptosis indicating that reactive oxygen species (ROS) may also play a major role [33]. Whether such inhibition of parasite cell death leads to hyperparasitism and vector killing has, however, not been elucidated. Together, these data show that apoptosis of procyclic trypanosomes in the midgut of its vector may contribute to the parasite density in this compartment but it is unknown whether this PCD is a form of self-restriction in order to facilitate transmission to new hosts or is rather a form to die after immune attack.

#### *Plasmodium *densities in the insect vector

Ookinetes of the rodent malaria species *Plasmodium berghei *can show several typical features of apoptotic cells both *in vivo *within the midgut of female anopheline mosquitoes and *in vitro *[34,35]. It should be stressed that the exact conditions that favour the occurrence of apoptotic *P. berghei *ookinetes *in vitro *still need to be unravelled since in another study only very low levels of ookinetes displaying typical features of apoptosis were detected [36]. Signs of apoptosis have recently also been found in *P. yoelli *ookinetes *in vitro *[37], and work published by Arambage and colleagues suggests that also *P. falciparum*, i.e. the etiological agent of human malaria tropica, can undergo apoptosis in the insect midgut [35]. *Plasmodium *ookinetes differentiate from zygotes after the uptake of macro- and microgametocytes with the blood meal from an infective host and fertilization. Since they subsequently develop within the midgut epithelium into oocysts with up to thousands of sporozoites, survival of ookinetes critically determines the parasitic load of the malaria vector. Importantly, distinct caspase inhibitors inhibit the occurrence of apoptotic *Plasmodium *midgut stages *in vitro *and *in vivo *and increase the number of oocysts significantly [34]. Caspases take a central role in apoptosis of higher eukaryotes but homologues are absent in protozoa [38]. It is, therefore, at first sight surprising that caspase inhibitors abolish apoptosis in *Plasmodium *parasites; however, this could be due to the unspecific inhibition of non-caspase parasite proteases by the high inhibitor concentrations used in that study. In common with other protozoan parasites, *Plasmodium spp. *express multiple proteases including cysteine proteases [39] that might help to disassemble the parasitic cell. In addition, metacaspases, i.e. related cysteine proteases that share with caspases the presence of a conserved catalytic dyad have been described in several protozoa including *Plasmodium falciparum *[8,38]. Whether they are indeed involved in the cell death of protozoa is debated since they also fulfil PCD-unrelated functions. Recently, clan CA cysteine proteases which include the cathepsins and calpains have been implicated in a chloroquine-induced apoptotic pathway in *P. falciparum *as determined by inhibitor studies [40]. While these studies provide valuable clues to possible cell death pathways, further functional analyses are clearly needed to prove a putative involvement of these proteases in the execution of protozoan apoptotic pathways. In should also be stressed that caspase-independent pathways of apoptosis are present in metazoans as well. Whatever the molecules involved, the results outlined above suggest that apoptosis in *Plasmodium *zygotes and/or ookinetes indeed regulate the intensity of infection within the *Anopheles *vector although alternative explanations as for example, an indirect effect via the modulation of apoptosis in insect host cells cannot be completely ruled out. As discussed for procyclic trypanosomes, it also awaits future clarification whether apoptosis of *Plasmodium *midgut stages has evolved as a form of self-restriction and is required in order to avoid death of the anopheline vector before transmission of the parasite to new hosts is accomplished.

Signs of apoptosis have also been observed in blood stages of *P. falciparum *[8,41-43] but this was not confirmed by others [7,9]. It thus has to be clarified under which conditions malaria blood stages exactly can initiate an apoptotic program. Also the hypothesis that such apoptosis may contribute to regulating the number of infected red blood cells [42] remains to be supported.

#### Regulation of *T. cruzi *densities inside and outside cells?

Markers of apoptosis have been extensively found in *T. cruzi*, i.e. the etiological agent of Chagas' disease, both *in vitro *and *in vivo *[44-47]. Exponential growth of axenic epimastigotes (EPI), i.e. the proliferative stage present in the reduviid vector leads to a massive increase of parasites with an apoptotic phenotype which is associated with onset of a stationary growth phase [44,47]. Importantly, the appearance of apoptotic cell death can be prevented by resuspending exponentially grown parasites at decreased densities and this also delays the onset of the stationary growth phase [44]. This clearly indicates that the appearance of apoptotic *T. cruzi *EPI depends on parasite density and may indeed regulate the size of the population *in vitro*. However, whether this also occurs in the reduviid vector and how this might be regulated is currently unknown.

In *T. cruzi*-infected cardiomyocytes, a varying proportion of intracellular amastigotes (AMA) can also undergo apoptosis *in vitro *and *in vivo *[45,46]. The regulated death of intracellular parasites is remarkable since a 'silent' removal of the apoptotic cells as described for mammalian cells or extracellular parasites cannot occur making the further fate of the dead cell questionable. Whatever the fate is, the parasite is nevertheless withdrawn from the pool of replicating AMA and may hence restrict the further propagation inside the host cell. This view is supported by findings that *in vitro *the numbers of intracellular AMA of two different *T. cruzi *strains inversely correlate with the number of TUNEL-positive parasites [46]. For example, ~50% of TUNEL-positive AMA of the *T. cruzi *clone Y (a type II biodeme) led to only 4-5 parasites per cardiomyocyte within two days of intracellular development whereas ~25% TUNEL positivity led to 10-18 parasites of the Dm28c clone (a type I biodeme) per host cell. This suggests a reduction of the intracellular infection level by 50-75% via an apoptotic cell death and provides a first hint that the level of *T. cruzi *AMA cell death might indeed contribute to the regulation of the intracellular level of infection [46]. However, one has to stress that time course analyses of prolongued infections with *T. cruzi *clone Y in cardiomyocytes did not corroborate this hypothesis since after three days of infection a significant increase of intracellular parasites was observed despite continuously high levels of TUNEL-positive AMA [48]. Thus, whether and under which conditions apoptosis in *T. cruzi *determines the level of intracellular infection awaits future clarification.

In conclusion, several findings including correlations between parasite densities and the occurrence of apoptotic parasites, the sensing of population sizes via distinct environmental cues, and the ability to experimentally manipulate parasite densities by altering cell death pathways clearly support the hypothesis that PCD in protozoa contributes to their density regulation. Clear experimental evidence to support this hypothesis has mostly been obtained *in vitro *whereas the situation *in vivo *is more complex and may often be obscured by the immune response of the host. Thus, while there is evidence that parasite PCD can indeed regulate parasite populations a main question that remains still unanswered is whether apoptosis in these parasites has evolved as a mechanism to determine parasite densities. Alternatively, apoptotic pathways in protozoan parasites can also be favoured during evolution by contributing to the evasion of the hosts' immune response thereby increasing parasite fitness.

### Immune silencing by apoptotic protozoan parasites

For the immune silencing potential of apoptotic protozoan parasites (Table 1) one has to take into account which lifestyle the parasite prefers. Being an obligate intracellular parasite preferring phagocytes as is the case for *Leishmania*, being intracellular in a wide range of host cells including phagocytic and non-phagocytic cells as is the case for *Toxoplasma *or a restricted range of non-phagocytic host cells as in the case of *Plasmodium *and *T. cruzi *as well as being extracellular as is the case for *T. brucei*.

Taking an obligate intracellular parasite preferring phagocytes, immune silencing of these host cells is a three step process [49]. This process is best described as the silent uptake of apoptotic mammalian cells into phagocytes [50-52]. First, apoptotic cells release "find-me" signals to recruit phagocytes to the site of apoptotic death [53]. Second, phagocytes recognize the presence of PS termed as "eat-me" signals on the membrane of apoptotic cells [51,54]. The final step is an active suppression of inflammation and immune response and can be termed as a "forget me" signal. Since apoptotic cells do not represent danger, their uptake does not result in the activation of antimicrobial effector functions of phagocytes [55,56]. This step is characterized by the release of anti-inflammatory cytokines such as TGF-β and IL-10 and lipids like the eicosanoids 15-S-HETE and lipoxin-A4 (LxA4). At the same time, pro-inflammatory cytokines like TNF and lipids like leukotriene-B4 are downregulated [50,52,57]. LxA4 enhances uptake of apoptotic cells and downregulates the production of IL-12 and the development of a TH-1 response [58].

#### Apoptotic *Leishmania*: An essential factor for successful infection

Taking *Leishmania *as a prototypic obligate intracellular parasite living inside phagocytes it has been demonstrated that *Leishmania *targets this three step process in at least two phases of establishing intracellular survival [49]. First *L. major *promastigotes produce a "find me" signal termed *Leishmania *chemotactic factor (LCF) recruiting its first host cell, i.e. the neutrophil (PMN) [59]. Moreover, it was shown that successful PMN invasion depends on the expression of the "eat me" signal PS on a sub-population of apoptotic parasites. After depleting the apoptotic parasites from a virulent population, *L. major *do not survive in phagocytes *in vitro *and lose their disease inducing ability *in vivo *[60,61]. It was also shown that promastigote survival depends on the PMN production of a "forget me" signal. PMN interaction with PS-positive promastigotes induce the production of TGF-β downregulating inflammatory TNF. Moreover, interaction with viable promastigotes alone induces a TNF dependent killing of intracellular promastigotes [60]. In the second phase of infection, *Leishmania *promastigotes infect macrophages and differentiate into the disease propagating multiplying amastigote form. Being first inside PMN these promastigote-infected cells start producing a "find me" signal in the form of MIP-1β specifically recruiting macrophages. At the same time infected PMN gradually becomes apoptotic and PS-positive. *L. major *can now use PMN as a 'Trojan horse' for a successful silent entry into macrophages [62,63]. As in the first phase of PMN infection this 'Trojan horse' strategy is mediated via the PS "eat me" signal on apoptotic PMN and "forget me" signals in the form of TGF-β produced by macrophages [62]. *In vivo *imaging has contributed a second evasion mechanism, recently termed 'Trojan rabbit' strategy, where parasites escape dying neutrophils to infect macrophages [63,64]. Disease propagation is mediated by amastigotes that were suggested to use PS expression as a form of apoptotic mimicry [65]. Here it was demonstrated that mouse-derived amastigotes are PS-positive and viable and that the presence of PS mediates a TGF-β dependent virulence of the amastigotes [65].

#### Immune silencing and trypanosomes

When analyzing the expression of PS on *T. cruzi *it was found that the apoptotic "eat me" signal was only present on the infective trypomastigotes (TRY), but not on the epimastigotes or intracellular amastigotes. In addition, it was demonstrated that the infective trypomastigotes uses a PS-dependent induction of TGF-β, thereby downregulating anti-parasitic activity of iNOS, enabling survival inside macrophages [66]. Moreover, *T. cruzi *has evolved a second evasion strategy based on the anti-inflammatory effect of TGF-β. In an experimental model for Chagas' disease it was found that the parasites induce an intense lymphocyte apoptosis [67]. Subsequently, this group demonstrated that the interaction of apoptotic but not necrotic lymphoyctes drives parasite growth in a TGF-β dependent manner [68]. Inhibiting the anti-inflammatory properties of TGF-β using cyclooxygenase inhibitors abolishes the pro-parasitic effect of apoptotic cell-macrophage interaction [68]. In a similar fashion injection of apoptotic neutrophils prior to *Leishmania *infection boost the parasitic growth [69].

As discussed above, *T. brucei *initiate cell death in the SS form which do not replicate and is unable to re-differentiate into replicating LS. The reason for this at first seemed unclear but it has been speculated that it constitutes a second control point after terminal differentiation [20]. Here, we speculate that it may rather represent a means to modulate the host's immune response to the parasite, since a continuously high number of stumpy parasites may favour an overwhelming inflammatory response with detrimental effects for both the parasite and the host. Apoptotic *T. brucei gambiense *have indeed been shown to dampen the inflammatory response of human macrophages [70].

#### Immune silencing and *Toxoplasma*

Inducible nitric oxide synthase (iNOS) regulation of nitric oxide (NO) also controls *T. gondii *growth. PS-expression on *T. gondii *was shown to induce TGF-β production by macrophages. The PS-binding protein Annexin-A5 reactivates the NO production and leads to the killing of *T. gondii *[71]. Therefore, an autocrine effect of TGF-β results in iNOS degradation, actin filament (F-actin) depolymerization and lack of nuclear factor-κB (NF-κB) in the nucleus contributing to PS-dependent *T. gondii *survival [71]. All these features can be reverted by TGF-β neutralizing antibody treatment. Recently, another mechanism involving PCD in the form of autophagy was shown to enhance intracellular *T. gondii *proliferation. *T. gondii *infection of both HeLa cells and primary fibroblasts induces host cell autophagy, dependent on ATG5 but independent of host mTOR signaling. Subsequently, this pathogen exploits the nutritive function of host autophagy to enhance its intracellular growth [72].

#### Immune silencing and *Plasmodium*

In contrast to the protozoan parasites mentioned above, for *Plasmodium *different hallmarks of apoptotic cell death have been recognized [35,73], but an immune silencing function has not yet been described. Since *Plasmodium *parasite stages do not need to immunologically silence phagocytes for a productive infection, it is not yet clear how PS-expression detected on different parasite stages can contribute in the parasites pathogenesis. Interestingly, the liver stage of the parasite hides inside a PS-negative bleb derived of hepatocytes to silently spread the parasites back into the blood circulation system [74].

### Autophagy, recycling of organelles, and parasite differentiation

Protozoan parasites possess complex life cycles and are regularly transmitted between completely different host species. The diverse environments of different hosts but also different life styles of the same parasite species within a given host require extensive parasite adaptations and differentiation. During recent years, autophagy in protozoa has emerged as crucial mechanisms during these processes (Table 1). Whether autophagy during parasite differentiation can also culminate in autophagic cell death, however, is still unclear.

#### Differentiation in *Leishmania *parasites

Database searches have identified homologues of autophagy related genes (ATG) in *Leishmania *parasites [75-77]. The coordinated action of ATG products regulates formation of autophagosomes, i.e. those organelles that contain the cellular cargo to be digested (see above). In both mammals and yeast, autophagosomes are formed by two different pathways: one involves ATG8, the other ATG12 and ATG5. Upstream of the autophagosome formation, TOR (target of rapamycin) kinases control cell growth in higher eukaryotes in response to nutrients, energy conditions, and growth factors, initiating or not autophagy. In *L. major*, all ATG proteins of the two ubiquitination cascades are present (reviewed by [12]). Functional complementation in *S. cerevisiae *ATG mutants demonstrated that homologues of ATG5, ATG8, ATG10 and ATG12 could replace their yeast counterparts [12].

It has been demonstrated that autophagy mediates differentiation in *L. major and L. mexicana *[75,76]. Based on GFP-labeled ATG8 that specifically label autophagosomes in *S. cerevisiae *it was demonstrated that an increase in the abundance of autophagosomes play a role in *L. mexicana *differentiation of procyclic promastigotes into metacyclics and their subsequent differentiation to disease propagating AMA [76]. In addition, it was demonstrated that cysteine peptidases CPA and CPB are essential in this process and can be blocked by the compound K11777 [76]. In mutant parasites without CPA and CPB, promastigotes have an enhanced number of autophagosome-containing lysosomes. Although these mutated parasites survived, stage differentiation into AMA was almost completely blocked. Moreover, it was demonstrated that by inactivation of the *L. major *VPS4 gene, late stage endosomal sorting was blocked resulting in an accumulation of cytosolic autophagosomes that could not be processed further. As a result, Lm-vps4 mutants were less able to survive under autophagy inducing conditions such as nutrient deprivation and these parasites were unable to differentiate into metacyclic promastigotes [75]. It has not yet been described whether autophagy in *Leishmania *can also result in PS-positivity and death.

#### Organelle recycling and differentiation in *T. brucei sp*

As other protozoan parasites, *T. brucei *has to adapt to completely different environments in different hosts. In mammals, LS parasites are adapted to the rich glucose environment of the blood [78], and they undergo rapid multiplication. Their energy metabolism depends exclusively on glycolysis, which takes place within glycosomes [79]. In contrast, SS parasites that accumulate at the peaks of parasitaemia do not divide, and activate at least some of the components required for fully active mitochondria as preadaptation to proline as the major energy source in the tsetse. After uptake with the tsetse blood meal, they complete the biochemical and morphological changes that culminate in the generation of a proliferative procyclic cell population in the vector midgut. Previous work shows that the metabolism and enzymatic contents of glycosomes but also their morphology and intracellular localization in BSF and procyclic forms differ considerably [80]. These studies revealed that old glycosomes were associated with lysosomes implicating microautophagy, and were finally completely disintegrated while new ones were built, resulting in a gradual shift [81]. Trypanosomes thus seem to have the machinery to specifically degrade glycosomes and probably mitochondria in a process called "glycophagy" [82]. These results confirm a role of autophagy in the differentiation and environmental adaptation of African trypanosomes.

#### Involvement of autophagy in *T. cruzi *differentiation

The most important triggers of differentiation of *T. cruzi *are high cell density and nutritional stress [83]. Cell density and nutritional stress are interconnected when the parasites reach stationary phase of growth in axenic cultures. Under these conditions, EPI undergo apoptosis (see above), although autophagy could be contributing to the increase in mortality during prolongued cultivation [47]. However, under conditions of serum deprivation, cytoplasmic vacuoles decorated with high concentrations of the ubiquitin-like ATG8.1, a characteristic feature of autophagy [47], can be observed. Mutation of the glycine residue at the C terminus of ATG8.1 abolishes accumulation, thus indicating processing by the cysteine peptidase ATG4 as observed in higher eukaryotes [84]. In *T. cruzi*, starvation of EPI occurs naturally in the gut of the insect vector, which is known to suffer long periods of lack of food (up to 12 months) [85]. Thus, autophagy could represent a crucial survival mechanism of *T. cruzi *in the gut of the insect vector.

During transformation of proliferating non-infective EPI into G0/G1-arrested metacyclic TRY (metacyclogenesis), EPI but not metacyclic TRY parasites massively die by a process resembling apoptosis (see above). This process also requires fast and extensive protein degradation and recycling of building blocks for the synthesis of new macromolecules. Recent results have shown an intense expression of ATG8.1 in differentiating EPI, suggesting that these cells are undergoing autophagy. In contrast, almost no ATG8.1 was expressed in normal EPI or in fully developed metacyclic TRY which suggests that autophagy is a very dynamic process [84].

Morphological and ultrastructural studies have shown that the main organelle that is transformed during metacyclogenesis are the reservosomes, the large endosomal compartments in EPI where proteins and lipids are accumulating [86]. Furthermore, reservosomes are absent in cell culture-derived AMA and TRY [86]. The reservosomal content is consumed during differentiation to metacyclics, when they shrink and finally disappear. Serine carboxypeptidase, i.e. a marker of reservosomes and ATG8.1 partially colocalize in the reservosomes during differentiation, a fact that is likely due to the delivery of the autophagosome content to the reservosomes/lysosomes [84]. These data strongly suggest that proteins accumulated in reservosomes are utilized by the EPI forms as energy source during differentiation and that autophagy may be crucial for their disappearance in the vertebrate stages.

### Autophagy and autophagic cell death as a response to stress

During their transmission between different host species or changes of their life style within a given host, parasites do not only undergo differentiation processes but they also encounter cellular stress, e.g. temperature shifts, starvation, or anti-parasitic effector mechanisms (Table 1). Autophagy has been recognized during environmental adaptations of several protozoan parasites which may facilitate parasite survival under certain stress conditions. As in higher eukaryotes, however, there is also in protozoan parasites a duality between pro-survival and death-promoting roles of autophagy. In addition, autophagic cell death in parasites has also been described under stress conditions although its function for the biology of the respective parasites remains elusive.

#### Stress adaptation in *T. brucei sp*

Starvation is a physiological condition that trypanosomes have to face within the insect gut. It has been shown that limited amount of nutrients can be transduced by the serine/threonine kinase TOR which is then inhibited, inducing autophagy. Starvation of *T. brucei in vitro*, by growing parasites in nutrient-limited medium or rapamycin, a macrolide isolated from *Streptomyces hygroscopicus *that binds to TOR, induce the formation of autophagic organelles [87]. It is questionable, however, if rapamycin induces autophagy in *T. brucei*, because it does not disrupt the active TOR complex as observed in higher eukaryotes [88].

ROS are common mediators of PCD which are increased after nutritional stress and after treatment with stress-inducing drugs [89]. In BSF trypanosomes, ROS are produced during prostaglandin-induced apoptosis (see above) although when it reaches higher concentrations can induce necrosis [25]. Remarkably, during these processes, autophagic structures can be seen in parasites that try to eliminate damaged structures and survive [87]. Likewise, dihydroxyacetone (DHA) that is used as a carbon source at low concentrations can lead to a cell cycle arrest in the G2-phase and formation of autophagic and multilamellar structures at higher concentrations [15,90]. This process is accompanied by cell membrane permeability, formation of ROS, PS exposure and cell death [15,90].

**Figure 1:**
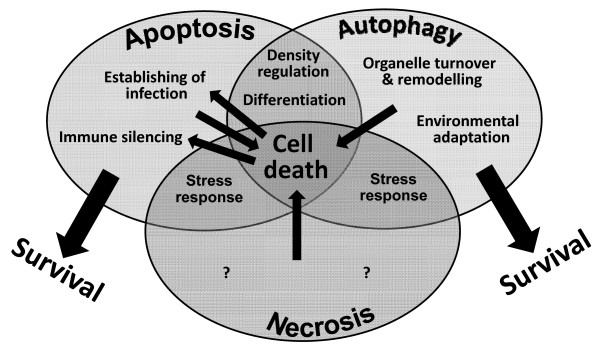
**Possible impact of protozoan PCD pathways in parasite biology and life-or-death decisions**. Parasitism depends on check points that control the population of the parasites and the survival of the host. In this sense, the existence of different PCD mechanisms in protozoan parasites appears to be crucial and may regulate distinct biological processes that are decisive for the outcome of infection. From a physiological point of view, PCD pathways can determine cell densities and differentiation at least under certain conditions, both processes being inter-connected by the regulated cross-talk between apoptotic and autophagic pathways. On the other hand, the involvement of autophagy on organelle remodelling allows the parasites to accomplish their complex life cycles and to adapt to different environments. Regarding the parasite-host interaction, several parasites are able to dampen immune responses and colonize the different environments in the hosts using apoptotic signalling. Deregulation of these processes can lead to activation of a predominant death signalling pathway that might be used as an attractive therapeutic strategy.

Neuropeptides have been recently identified in mammals after *T. brucei *infection. These peptides are targeted to the parasite glycosome and induce autophagic cell death in BSFs but not procyclic forms [16]. Neuropeptide-mediated autophagic cell death in *T. brucei *is preceeded by an energy metabolism failure and can thus be considered as being stress-related. Although the effect of neuropeptides against LS or SS has to be determined, this is an example of density control that could also contribute to parasite differentiation (Figure 1; see also above). Differences between structure and abundance of glycosylphosphatidylinositol (GPIs), glycosylation patterns of surface proteins of BSFs and procyclic forms, different endocytosis rates, carbohydrate metabolism, and glycosome dependency presented by each stage may be involved in the differential susceptibility of different life-cycle stages to these molecules.

#### Autophagy in the stress response of *T. cruzi*

Treatment of *T. cruci *with lysophospholipid analogues such as edelfosine or miltefosine leads to cytosolic membrane arrangements, mitochondrial swelling and appearance of concentric structures in EPI, i.e. the insect stage, resembling autophagosome-like structures [17]. Naphthoquinones derived from plants lead to signs of an apoptosis-like process but formation of atypical membrane structures in EPI and trypomastigotes (TRY) suggests also the involvement of autophagy [18]. Propolis, a bee product with microbicidal properties causes mitochondrial swelling in EPI, with new membrane structures inside this organelle and, in the cytosol, altered vacuoles, formation of myelinated structures, and reservosome disorganization [91]. This suggests a possible interference with lipid content and biosynthesis, and in macromolecules accumulation [92]. Using terpenic alcohols such as geranylgeraniol, endoplasmic reticulum disorganization, myelin-like structures and concentric membrane arrangements inside damaged mitochondria were observed in all stages of *T. cruzi*, suggesting autophagic events [93]. The ultrastructure of the parasites after treatment with these chemotherapeutic agents suggests autophagic cell death as a common phenotype although signs of necrosis and apoptosis were also observed [6]. This suggests the interplay of distinct death mechanisms through a cross-talking of signalling pathways as reported for mammalian cells (Figure 1) [94]. Recently, the molecular features of autophagy in *T. cruzi *have been characterized [84]. A bioinformatic analysis of the *T. cruzi *genome confirms the existence of all major genes of the ATG8 conjugation system (ATG3, ATG4, ATG7, ATG8), whereas the major components of the ATG12-ATG5 conjugation system (ATG12, ATG5, ATG10) are apparently lacking [95]. The reduced set of genes involved in regulation and signalling pathways of autophagy in trypanosomes as compared to other eukaryotes might reflect the early appearance of this process in evolution [96]. The two *T. cruzi *autophagins (ATG4 proteases) are expressed constitutively throughout the parasite life cycle and process two recombinant ATG8 homologues at the Gly residue [84]. Moreover, all the *T. cruzi *ATG4, and to a lesser extent ATG8 homologues substitute their yeast counterparts in functional assays [84]. These results show that ATG8 conjugation system in *T. cruzi *is highly similar to the mammalian one, suggesting evolutionary conservation of this autophagic pathway.

## Conclusions

Over the past 10-15 years considerable progress has been made in the understanding how distinct PCD pathways in protozoan parasites may affect the biology of protozoan parasites and their interactions with mammalian hosts and invertebrate vectors (Figure 1). The appearance of parasites displaying markers of apoptosis or autophagy during distinct processes of parasite biology as well as functional evidences has fuelled the view that these PCD pathways play critical roles in the life style of parasitic protozoa. In contrast, the impact of parasite necrosis for parasite-host interactions and whether it can occur in a regulated fashion as described in metazoans [10,11] is completely unknown. There is now good evidence that densities of distinct trypanosomatids and apicomplexan parasites can correlate with the appearance of PCD markers indicative of apoptosis. It has to be stressed, however, that a definite proof for the concept of parasite density regulation by parasite apoptosis is still missing, especially *in vivo*. This will probably require the availability of mutant parasites that are deficient in distinct regulators or executors of apoptotic cell death indicating also the urgent need to characterize underlying molecular mechanisms of PCD in protozoan parasites. There are also clear indications that apoptotic parasites with PS present in the outer leaflet of their plasma membrane modulate host immunity by limiting the inflammatory response of the host. This can be viewed as a form of molecular mimicry by which parasites 'misuse' a pathway for the immunologically silent removal of PS-exposing cells of their mammalian hosts. Another common theme emerging from the examples discussed above is that autophagy is involved in the differentiation of protozoan parasites. In addition, autophagy and autophagic cell death appear to be common responses when parasites encounter environmental stress. Since differentiation often coincides with environmental stress, for example during transmission from mammalian hosts to vectors or vice versa the contribution of autophagy and/or autophagic cell death in these processes may not be clearly distinguishable in each case. Nevertheless, is has become clear that autophagy in protozoan parasites - as in metazoan - primarily promotes survival but can proceed to a programmed form of death if adverse conditions within a hostile environment exceed cellular capacity. Although we are certainly far away from a clear understanding, apoptosis and autophagy thus appear to play important roles in protozoan parasites and the interactions with their mammalian hosts and invertebrate vectors. A detailed knowledge of the underlying molecular mechanism might open the possibility to combat protozoan parasites efficiently by promoting their own death pathways.

## Competing interests

The authors declare that they have no competing interests.

## Authors' contributions

CL collected data and drafted the information on the occurrence of PCD in protozoan parasites and its impact on cell density regulation. He also coordinated the contributions of the different authors. JC-S collected data and contributed to the comparing of PCD in protozoan parasites and higher eukaryotes. EG-R collected data on autophagy and its impact for the course of infection and drafted the respective part of the manuscript. GvZ collected data on immune silencing by apoptotic protozoa and drafted the respective part of the manuscript. EG-R and GvZ contributed equally to this work. All authors read and approved the final manuscript.
